# Supplementary material for: Uncovering the mechanism linking education expectation and suicide ideation among students in tertiary education: The mediating role of academic burnout, psychache, and hopelessness
Source: PLoS One. 2025 Dec 18;20(12):e0337831. doi: 10.1371/journal.pone.0337831 (PMC12714256; doi:10.1371/journal.pone.0337831)
Supplement: Attachment — Submitted filename: Response to Reviewers.docx [file pone.0337831.s004.docx]

**RESPONSE TO EDITOR**

1. Please make the objective of your study clear and easily understandable. It is not clear what you are going to do from your title?

We have revised the title to clearly indicate the objective of the study. The new title reads: *Uncovering the mechanism linking educational expectations and suicide ideation among students in tertiary education: The mediating role of academic burnout, psychache, and hopelessness.*

We have also revised the section on the Current Study (on line 140) to better clarify the aim of the study for ease of understanding.

2. Under introduction it is better to start with definition and burden of the problem world wide than of the specific country.

We believe we have sufficiently provided the burden of the problem regarding youth and student suicide on a global scale (i.e., Asian continent, Western Pacific) on line 48. To further support this, we have added details and a definition of suicide to the first paragraph as well.

3. Your result and methodology seems to have no clear boundary. Some bio-demographic data are the under methodology section.

Additional details regarding the inclusion criteria of the sample has been included (see line 166). This highlights the boundary regarding our participant selection. We opted to report demographic data in the Participant sub-section to allow the Result section to focus solely on the analysis of the hypothesized model.

4. The major finding of the study should be included in the conclusion section than only recommending researchers.

We have revised the conclusion and include a short summary of the major findings (see line 461).

5. Some other components like abbreviations,... could be included in the document.

We acknowledge the comment and feel that the current amount of abbreviations is sufficient to aid understanding of the manuscript’s content.

We would like to thank the editor for the comments and guidance provided to the manuscript.

**RESPONSE TO REVIEWERS**

Reviewer's Responses to Questions

Comments to the Author

1. Is the manuscript technically sound, and do the data support the conclusions?

The manuscript must describe a technically sound piece of scientific research with data that supports the conclusions. Experiments must have been conducted rigorously, with appropriate controls, replication, and sample sizes. The conclusions must be drawn appropriately based on the data presented.

Reviewer #1: Yes

Reviewer #2: Yes

Thank you

2. Has the statistical analysis been performed appropriately and rigorously?

Reviewer #1: I Don't Know

Reviewer #2: Yes

Thank you

3. Have the authors made all data underlying the findings in their manuscript fully available?

The PLOS Data policy requires authors to make all data underlying the findings described in their manuscript fully available without restriction, with rare exception (please refer to the Data Availability Statement in the manuscript PDF file). The data should be provided as part of the manuscript or its supporting information, or deposited to a public repository. For example, in addition to summary statistics, the data points behind means, medians and variance measures should be available. If there are restrictions on publicly sharing data—e.g. participant privacy or use of data from a third party—those must be specified.

Reviewer #1: Yes

Reviewer #2: Yes

Thank you

4. Is the manuscript presented in an intelligible fashion and written in standard English?

PLOS ONE does not copyedit accepted manuscripts, so the language in submitted articles must be clear, correct, and unambiguous. Any typographical or grammatical errors should be corrected at revision, so please note any specific errors here.

Reviewer #1: Yes

Reviewer #2: Yes

Thank you

5. Review Comments to the Author

Please use the space provided to explain your answers to the questions above. You may also include additional comments for the author, including concerns about dual publication, research ethics, or publication ethics. (Please upload your review as an attachment if it exceeds 20,000 characters)

Reviewer #1: This subject is of high importance due to the recent increased attempts and ideation of suicide by the students, globally. I particularly liked the examining of the underlying mechanisms and relations between multi factors.

Thank you

Reviewer #2: This is a very interesting study and has implications for both educators and counsellors who work with students, along with curriculum designers. Although the authors have presented data regarding the sources of expectations on students, it was not clear if these actually led to suicidal ideation or if that was assumed or implied by the authors without supportive substantive data. Kindly clarify this aspect of your study.

The study attempts to provide more insight into the narrative that high educational expectations can lead to suicidal outcomes among student (see line 80 to 85). In this study, we argue that high educational expectations do not necessarily lead to suicide ideation and that the link is more nuanced. We proposed that academic burnout, psychache, and hopelessness are mediating factors that can explain variability in the risk of suicide ideation due to high educational expectation (see line 140 onwards).

Data about suicidal ideation among students were collected using the Suicide Ideation Scale (see line 199). This scale provides data on the degree of suicidal ideation with higher scores indicating greater suicidal ideation than lower scores. Our analysis through mediation modelling, tests the predictability of educational expectation on suicide ideation by examining the influence of the mediators (i.e., academic burnout, psychache, hopelessness). In all our findings, the data suggests that educational expectations do not *directly* predict suicide ideation and that the link between educational expectations and suicide ideation varies according to the source of expectation and through mediating factors (see Results section).

6. PLOS authors have the option to publish the peer review history of their article (what does this mean?). If published, this will include your full peer review and any attached files. If you choose “no”, your identity will remain anonymous but your review may still be made public. Do you want your identity to be public for this peer review? For information about this choice, including consent withdrawal, please see our Privacy Policy.

Reviewer #1: Yes: Dr Lily Abedipour MD

Reviewer #2: No

Thank you

We would like to thank the reviewers for their time and feedback provided to the manuscript.

**JOURNAL REQUIREMENTS**

1. The manuscript has been revised to meet the style requirements for PLOS ONE.
2. We are unclear about this comment as we have indicated that, in the Additional Information page of the Submission Form, under Data Availability that our **data is fully available without restriction** and provided a link to where the data can be found.

In the following box (**Describe where the data may be found in full sentences**), we stated that the data supporting the conclusions of the article can be found in the Research Data repository using the link <https://research.jcu.edu.au/data/published/8bbaba10d57e11ee982481db70fb3be2>.
The data is currently under review and will be made publicly available.

Please advise should this be insufficient.

3. Captions for each figure has been included in the manuscript.
4. We have revised the reference list according to the correct format. We have updated several references with more recent links and have checked that none of the articles have been retracted. We have also made a change to reference as follows:
   a. Ref no [3] from Fuse-Nagase et al. (2021) to De Paula et al. (2020) based on the recommendations of the editor to provide additional support to the introduction.
   b. Ref no [57] from Au (1995) to Au et al. (2009) to ensure that links are still available.

We have revised the manuscript to our best to meet the requirements of the journal and address the comments made by the editor and reviewers. We hope that the revised manuscript will be considered for publication by the Journal and will be happy to address and provide any information necessary. Thank you.
