# Supplementary material for: Uncovering the mechanism linking education expectation and suicide ideation among students in tertiary education: The mediating role of academic burnout, psychache, and hopelessness
Source: PLoS One. 2025 Dec 18;20(12):e0337831. doi: 10.1371/journal.pone.0337831 (PMC12714256; doi:10.1371/journal.pone.0337831)
Supplement: Attachment — Submitted filename: Review for PLOS ONE.docx [file pone.0337831.s002.docx]

**Review for PLOS ONE**

Thank you for your hard work and devotion to this important study. This work is clearly the result of extensive research, and it provides useful knowledge into the complex relationship between educational expectations and suicide ideation among tertiary students. However, a few issues need to be solved in order to improve the study's clarity, depth, and overall significance.

1. This statistic is outdated. Try to find updated data from around 2020 to ensure relevance and accuracy. For example, World Health Organization database had a suicide rate of 12.6 per 100,000 among 15 to 29-year-olds in the Western Pacific region in 2008.
2. Although the introduction provides a global overview of youth suicide statistics, the transition to the core research question could be more specific. The current narrative should better highlight how this study fills a gap in understanding the specific mechanisms linking educational expectations to suicidal ideation. Refine the research question to clearly emphasize why investigating these mediators (academic burnout, psychache, and hopelessness) in this context is novel and valuable.
3. The literature review, although informative, should engage more with existing research on academic stressors and suicide risk. Make sure there is a clear connection between past studies and the rationale for selecting academic burnout, psychache, and hopelessness as mediators. Integrate more theoretical backing or discuss prior findings on these mediators to justify their selection.
4. The methodology is sufficiently detailed, but the use of convenience and snowball sampling may limit the generalizability of the findings. Acknowledge this as a limitation and suggest strategies for future studies to employ more representative sampling techniques. Clarify the ethical measures taken to ensure participant safety, especially considering the study's sensitive nature.
5. There is no mention of how missing data was handled, other than omitting incomplete responses, which could introduce bias. Include a brief discussion on whether imputation methods were considered for handling missing data and the potential impact of their omission on the results.
6. The scales used in this study show varied levels of internal consistency. Although the Psychache Scale has strong reliability (α = .92), the Higher Educational Expectation Scale (α = .70) is on the lower end of acceptability. Discuss the implications of employing instruments with different reliability levels and suggest comparing findings with alternative measures in future studies to reinforce conclusions.
7. The manuscript does not provide information on whether the scales were validated for use in multicultural contexts, such as Malaysia, Singapore, and Australia. Justify the choice of these instruments and note if any cross-cultural adaptations were made. Recommend future studies to conduct or include such validations to enhance cross-cultural reliability.
8. The authors applied structural equation modeling (SEM) to examine their hypothesized model; however, the rationale for specifying 12 separate models is unclear. Provide an explanation of how each model contributes to the broader understanding of the research question. Include a discussion on the criteria and determination of model fit indices (e.g., RMSEA, CFI, TLI) to ensure thorough reporting.
