# Supplementary material for: Uncovering the mechanism linking education expectation and suicide ideation among students in tertiary education: The mediating role of academic burnout, psychache, and hopelessness
Source: PLoS One. 2025 Dec 18;20(12):e0337831. doi: 10.1371/journal.pone.0337831 (PMC12714256; doi:10.1371/journal.pone.0337831)
Supplement: Attachment — Submitted filename: reviewer3.docx [file pone.0337831.s003.docx]

PLOS ONE
Uncovering the mechanism linking education expectation and suicide ideation among
students in tertiary education: The mediating role of academic burnout, psychache, and
hopelessness.
--Manuscript Draft—

- In lines 22 to 28 of the abstract, the following points should be noted:

It seems that the role of mediator and moderator should be modified as follows:

Mediator factor:

academic burnout, (It has an indirect effect on behavior)

Moderator factors:

Psychache and hopelessness:

) It has a direct effect on the behavior and the relationship between high educational expectations and suicide ideation(

- In other words, here it is better to once again calculate the direct effect of psychache and

hopelessnesson on the relationship between high academic expectations and suicide ideation in the role of moderator.

It should be determined how much psychache and hopelessnesson are related to academic burnout and education expectation, because these factors may be related to personality factors and other factors that moderate the relationship here.

- At the end of the introduction (line 73), attention should be paid to the following points:

Considering that the study sought to discover mediators and their effect in the relationship between education expectation and suicide ideation, the role of mediators should be mentioned here.

- According to the method section, the participants section (lines 168 to 175):

There is a possibility of attrition bias. It should be mentioned.

- According to the graphs (Hypothesized serial mediation model), the role of moderators has not been taken into account in relation to variables. If possible, it is suggested as follows:

Possible biases in the study should be mentioned as limitations:

Temporality bias : Psychache and Hopelessness may exist before high academic expectations emerge

Information bias: Questionnaires measuring Psychache and Hopelessness should be specifically related to the variable of high educational expectations. It seems that a general questionnaire was used here.

attrition bias: According to the method section, the participants section (lines 168 to 175)

RESPONSE TO REVIEWERS
1. Is the manuscript technically sound, and do the data support the conclusions?
The manuscript must describe a technically sound piece of scientific research with data
that supports the conclusions. Experiments must have been conducted rigorously, with
appropriate controls, replication, and sample sizes. The conclusions must be drawn
appropriately based on the data presented.
Reviewer #1: Yes
Reviewer #2: Yes

Reviewer #3:Yes
Thank you
2. Has the statistical analysis been performed appropriately and rigorously?
Reviewer #1: I Don't Know
Reviewer #2: Yes

Reviewer #3:Yes
Thank you
3. Have the authors made all data underlying the findings in their manuscript fully
available?
The PLOS Data policy requires authors to make all data underlying the findings
described in their manuscript fully available without restriction, with rare exception
(please refer to the Data Availability Statement in the manuscript PDF file). The data
should be provided as part of the manuscript or its supporting information, or deposited
to a public repository. For example, in addition to summary statistics, the data points
behind means, medians and variance measures should be available. If there are
restrictions on publicly sharing data—e.g. participant privacy or use of data from a third
party—those must be specified.
Reviewer #1: Yes
Reviewer #2: Yes

Reviewer #3:Yes
Thank you
4. Is the manuscript presented in an intelligible fashion and written in standard
English?
PLOS ONE does not copyedit accepted manuscripts, so the language in submitted
articles must be clear, correct, and unambiguous. Any typographical or grammatical
errors should be corrected at revision, so please note any specific errors here.
Reviewer #1: Yes
Reviewer #2: Yes

Reviewer #3:Yes
Thank you
5. Review Comments to the Author
Please use the space provided to explain your answers to the questions above. You
may also include additional comments for the author, including concerns about dual
publication, research ethics, or publication ethics. (Please upload your review as an
attachment if it exceeds 20,000 characters)
Reviewer #1: This subject is of high importance due to the recent increased attempts
and ideation of suicide by the students, globally. I particularly liked the examining of the

underlying mechanisms and relations between multi factors.
Thank you
Reviewer #2: This is a very interesting study and has implications for both educators
and counsellors who work with students, along with curriculum designers. Although the
authors have presented data regarding the sources of expectations on students, it was
not clear if these actually led to suicidal ideation or if that was assumed or implied by
the authors without supportive substantive data. Kindly clarify this aspect of your study.
The study attempts to provide more insight into the narrative that high educational
expectations can lead to suicidal outcomes among student (see line 80 to 85). In this
study, we argue that high educational expectations do not necessarily lead to suicide
ideation and that the link is more nuanced. We proposed that academic burnout,
psychache, and hopelessness are mediating factors that can explain variability in the
risk of suicide ideation due to high educational expectation (see line 140 onwards).
Data about suicidal ideation among students were collected using the Suicide Ideation
Scale (see line 199). This scale provides data on the degree of suicidal ideation with
higher scores indicating greater suicidal ideation than lower scores. Our analysis
through mediation modelling, tests the predictability of educational expectation on
suicide ideation by examining the influence of the mediators (i.e., academic burnout,
psychache, hopelessness). In all our findings, the data suggests that educational
expectations do not directly predict suicide ideation and that the link between
educational expectations and suicide ideation varies according to the source of
expectation and through mediating factors (see Results section).

Reviewer #3:The study attempts to provide more insight into the narrative that high educational expectations can lead to suicidal outcomes among student, But it seems that the study tools should specifically measure the amount of mediating factors caused by high academic expectations. Also, it has not determined the role of other factors such as personality factors that affect the amount of these mediating factors.

Thank you
6. PLOS authors have the option to publish the peer review history of their article (what
does this mean?). If published, this will include your full peer review and any attached
files. If you choose “no”, your identity will remain anonymous but your review may still
be made public. Do you want your identity to be public for this peer review? For
information about this choice, including consent withdrawal, please see our Privacy
Policy.
Reviewer #1: Yes: Dr Lily Abedipour MD
Reviewer #2: No

Reviewer #3:No
Thank you
