# Supplementary material for: Uncovering the mechanism linking education expectation and suicide ideation among students in tertiary education: The mediating role of academic burnout, psychache, and hopelessness
Source: PLoS One. 2025 Dec 18;20(12):e0337831. doi: 10.1371/journal.pone.0337831 (PMC12714256; doi:10.1371/journal.pone.0337831)
Supplement: Attachment — Submitted filename: Response to Reviewers_PLOS ONE.docx [file pone.0337831.s005.docx]

**RESPONSE TO REVIEWERS FOR PLOS ONE**

We would like to thank the reviewer for the feedback and response towards the manuscript. Our response to the comments is as follows (in blue).

1. This statistic is outdated. Try to find updated data from around 2020 to ensure relevance and accuracy. For example, World Health Organization database had a suicide rate of 12.6 per 100,000 among 15 to 29-year-olds in the Western Pacific region in 2008.

The statistics have been updated with the line as follows:

World Health Organization database revealed a suicide rate of 17.18 per 100,000 among 15 to 29-year-olds in the Western Pacific region based on data collected in 2021 [5].

The citation has also been updated accordingly.

2. Although the introduction provides a global overview of youth suicide statistics, the transition to the core research question could be more specific. The current narrative should better highlight how this study fills a gap in understanding the specific mechanisms linking educational expectations to suicidal ideation. Refine the research question to clearly emphasize why investigating these mediators (academic burnout, psychache, and hopelessness) in this context is novel and valuable.

A revision that highlights how the study addresses the gap through the mediators has been added as follows:

Youth suicide is preventable and insight into the mechansims of suicide development is necessary to inform research and prevention. It is clear that high expectations can lead to negative psychological outcomes [19,25,26] which are manifestations of psychological pain and a sense of hopelessness [45,46]. We also identified academic burnout as both an outcome of high educational expectations (i.e., a demand beyond a student’s control) and a predictor of suicide ideation per the JDC [38]. As academic burnout is characterized by feelings of emotional exhaustion, cynicism, and inadequacy, we propose that academic burnout also predicts psychache and hopelessness which subsequently predicts suicidal ideation. Cynical beliefs about the purpose of studying along with the perceived incompetence as a student signify a pessimistic outlook and a sense of hopelessness toward their academic pursuit [16]. As such, the present study proposes a novel model that integrates the 3ST and the JDC in an attempt to examine the mechanism between educational expectations and suicide ideation through hypothesized a serial mediation model. Through this model, we empirically identified how burnout, psychological pain, and hopelessness predicts suicide risk with the goal of informing targeted suicide prevention in mitigating the burden of educational expectations among students in tertiary education.

3. The literature review, although informative, should engage more with existing research on academic stressors and suicide risk. Make sure there is a clear connection between past studies and the rationale for selecting academic burnout, psychache, and hopelessness as mediators. Integrate more theoretical backing or discuss prior findings on these mediators to justify their selection.

We believe that we have succinctly drawn connections between academic expectations and suicide risk by utilizing the Three-step theory of Suicide and the Job Demand Control model to fit within the journals word limit. The selection of mediators was informed by these models where i) psychache and hopelessness was drawn from the development of suicide ideation proposed in the 3ST and ii) burnout as a mediator between high demand and poor psychological outcomes as proposed by the Demand control model. We have noted this in The Present Study section of the manuscript.

4. The methodology is sufficiently detailed, but the use of convenience and snowball sampling may limit the generalizability of the findings. Acknowledge this as a limitation and suggest strategies for future studies to employ more representative sampling techniques. Clarify the ethical measures taken to ensure participant safety, especially considering the study's sensitive nature.

An acknowledgment of the sampling limitations was added in the Limitations section as follows:

Given the sampling limitations, caution should be taken in generalizing the findings. Future research should utilize a more representative technique to sample university student populations.

Additionally, an explanation about the ethical steps taken to ensure participant safety was added in the Methods section as follows:

Given the sensitive nature of the study, participants were made aware in the Information document about the risks of distress and were provided with contact details of free psychological support services in respective countries in the support document.

5. There is no mention of how missing data was handled, other than omitting incomplete responses, which could introduce bias. Include a brief discussion on whether imputation methods were considered for handling missing data and the potential impact of their omission on the results.

We have described in the Method section that incomplete or missing responses were omitted from the dataset prior to analysis. As such, no further imputation methods were considered as these missing cases were removed. We added a sentence in the Results to highlight this as follows:

Prior to analysis, the dataset was screened. Any incomplete responses were treated as missing completely at random and dropped from the final dataset. Note that possible bias may occur due to deletion by not accounting for reasons for missingness.

6. The scales used in this study show varied levels of internal consistency. Although the Psychache Scale has strong reliability (α = .92), the Higher Educational Expectation Scale (α = .70) is on the lower end of acceptability. Discuss the implications of employing instruments with different reliability levels and suggest comparing findings with alternative measures in future studies to reinforce conclusions.

To address this, we have included the reliability scores for each scale based on the data from this study across the three samples in the Method section. A summary for your reference is as follows:

| Instrument | Malaysia | Singapore | Australia |
| --- | --- | --- | --- |
| HEES | .905 | .912 | .910 |
| Psychache | .958 | .952 | .957 |
| BHS | .841 | .841 | .828 |
| SBI | .886 | .873 | .886 |
| SIS | .922 | .938 | .945 |

Overall, the reliability scores were strong (>.80) for all.

7. The manuscript does not provide information on whether the scales were validated for use in multicultural contexts, such as Malaysia, Singapore, and Australia. Justify the choice of these instruments and note if any cross-cultural adaptations were made. Recommend future studies to conduct or include such validations to enhance cross-cultural reliability.

A description of the limitation regarding cross-cultural validation of the scale was added as follows:

Furthermore, cross-cultural validation and reliability of the instruments used in this study across Malaysia, Singapore, and Australia needs to be examined further.

8. The authors applied structural equation modeling (SEM) to examine their hypothesized model; however, the rationale for specifying 12 separate models is unclear. Provide an explanation of how each model contributes to the broader understanding of the research question. Include a discussion on the criteria and determination of model fit indices (e.g., RMSEA, CFI, TLI) to ensure thorough reporting.

We have revised the Proposed analysis section to provide more details on the specification of the model to explain how analysis were conducted.

The study employed a structural equation modelling with the maximum likelihood estimation using the Statistical Package for Social Science 27.0 AMOS 28. We specified the model with one exogenous variable (educational expectations), four endogenous variables (academic burnout, psychache, hopelessness, suicide ideation), and nine pathways based on the postulations by the 3ST and JDC theory resulting in an over-identified model (Fig 2). The same form (model) was tested with data for each source of educational expectation (self, parents, educator/institution, culture) respectively while data for the other variables remained constant. Testing the model for the different sources of expectations allows us to better identify how each source influences poor mental health outcomes. Multigroup analysis was performed in AMOS 28 on each source of expectation model with Group 1 (Malaysia), Group 2 (Singapore), and Group 3 (Australia). Estimates of model fit for each model include the comparative fit index (CFI), normed fit index (NFI), and goodness-of-fit statistic (GFI) values near or greater than 0.95, and a root mean square error of approximation (RMSEA) value near or less than 0.06 [52].

A pairwise parameter comparison using the critical ratio for differences on the nine specified pathways was examined. For a two-tailed test, a critical ratio difference z-score between the critical range of ±1.96 indicates no significant difference. Multigroup analysis allows us to determine cross-cultural variances on the effect of different types of expectations on poor mental health outcomes.

We have also restructured the Serial mediation analysis sub-section in the Results section to make it easier to describe the findings for each model and the model fit. Model fit indices were added for each model accordingly. We have reorganized the figures accordingly.

General response.

We sincerely appreciate the feedback and suggestions provided by Reviewer 3 regarding our manuscript. Suicide is a complex issue research on trying to comprehend what predicts an increased risk of suicide is highly debatable but vital. Multiple perspectives and theories exist in attempts to illuminate the underlying mechanisms in the development of suicidal outcomes. For our study, we specifically focused on addressing and providing insight into the link between high educational expectations and suicide ideation among students in higher education. Here, we utilized the 3ST and the JDC model to examine the mechanism linking expectations and suicide ideation development. We focused on academic expectation as a form of demand, leading to poor psychological outcomes such as academic burnout, psychache, and hopelessness (mediators), which in turn increases the risk of suicide ideation based on the postulations in these theoretical models. As such, we did not test other variables, such as personality, in this study. We have acknowledged that future research, however, should consider the role of other factors that may influence the pathways between expectations and suicide ideation. Given the theoretical postulations, we also did not examine burnout, psychache, and hopelessness as moderators, though we acknowledge that future investigation may examine how different levels of psychache and hopelessness may moderate the link between academic expectation, academic burnout, and suicidal ideation among students. The suggestions given by Reviewer 3 are valuable input for suicide research, and we will continue to pursue our future investigations through a more comprehensive model. We would like to thank the reviewers for their time and input.
