# Supplementary material for: Uncovering the mechanism linking education expectation and suicide ideation among students in tertiary education: The mediating role of academic burnout, psychache, and hopelessness
Source: PLoS One. 2025 Dec 18;20(12):e0337831. doi: 10.1371/journal.pone.0337831 (PMC12714256; doi:10.1371/journal.pone.0337831)
Supplement: Attachment — Submitted filename: Response to Reviewers - 24.10.2025.docx [file pone.0337831.s006.docx]

**RESPONSE TO REVIEWERS**

Review Comments to the Author and response;

Reviewer #4: Thank you for your valuable contribution to the field. The topic is timely and relevant, and it is clear that significant effort went into data collection across multiple countries. This review focuses primarily on the Method section, which is foundational to the overall quality of the research. Several areas require clarification:

Thank you for your time and feedback on the manuscript. We have provided responses to your comments in the following (in blue).

1. In the method section, the % of gender identification in the Malaysian sample do not add up to 100% (77.7% female + 21% male + 13% unidentified = 111.7%)

Thank you for highlighting this error. It appears we have missed the decimal point for the unidentified group, which was supposed to be 1.3% not 13%. This has been corrected.

See line 171, page 8 of Tracked Manuscript

2. “Unidentified” is vague—does this refer to participants who chose not to disclose, non-binary participants, or missing data?

A revised wording to better reflect the option of not disclosing (i.e., prefer not to say) has replaced the word ‘unidentified’ in the manuscript.

See lines 174, 176, and 178, page 8 of Tracked Manuscript

3. Explain the criteria for excluding responses due to missing data. Mention if any imputation methods were considered.

We have explained this process in the Preliminary Analysis section. Here, we noted that during data screening, incomplete responses were treated as missing and dropped from the final dataset. We also added that ‘… no imputation methods used…’.

See lines 250 – 251, page 12 of Tracked Manuscript.

4. The section does not justify why the final sample sizes (224, 200, 217) are adequate for the analysis.

An additional statement was provided as follows:

… The final sample size for each country was adequate for a moderate effect size in regression models (Wolf et al., 2013).

See lines 179 – 180, page 8 of Tracked Manuscript.

The reference list has also been updated to include Wolf et al. (2013).

5. “At the time of data collection” is vague. The specific time period (e.g., semester, month/year) should be mentioned.

A revised sentence that clearly mentioned the data collection period was added as follows:

… between July 2022 and December 2022.

See line 171, page 8 of Tracked Manuscript.

6. There is no mention of ethics approval from any Institutional Review Board (IRB) or Ethics Committee

A statement was added to the manuscript as follows:

… approved by the James Cook University Human Research Ethics Committee (H8552).

See line 225 – 2256, page 11 of Tracked Manuscript.

7. What language(s) the survey was administered in? How long did it take participants to complete the survey?

Additional details were added to the manuscript as follows.

The 15-minute study was in English…

See line 225, page 11 of Tracked Manuscript.
